# The effects of short term intravenous infusion of a soybean based lipid emulsion on some blood constituents in sheep: A preliminary study

**Published:** 2014

**Authors:** Hamid Akbari, Bahram Dalir-Naghadeh

**Affiliations:** Department of Clinical Sciences, Faculty of Veterinary Medicine, Urmia University, Urmia, Iran

**Keywords:** Blood constituents, Insulin resistance, Lipid emulsion, Sheep

## Abstract

To evaluate the effect of intravenous infusion of a soybean based lipid emulsion (Lipovenoes 10%) on some blood constituents in sheep, a replicated 2 × 2 Latin square design experiment was conducted in four clinically healthy ewes. Lipid emulsion (LE group) or normal saline (NS group) was infused intravenously at a rate of 0.025 mL kg^-1^ per min for 6 hr and the concentrations of blood triglyceride, glucose, insulin, calcium, magnesium, phosphorous, sodium and potassium were measured before (baseline) and then at timepoints 2, 4, 6, 12 and 24 hr after infusion. Compared to the baseline values and/or NS infusion, LE infusion resulted in a significant increase in the concentrations of triglyceride (*p* < 0.001), glucose (*p* < 0.01), calcium (*p* < 0.05), phosphorous (*p* < 0.01) and a significant decrease in insulin (*p* < 0.001) and magnesium (*p* < 0.05) concentrations. Compared to the baseline value, the homeostasis model of insulin resistance (HOMA-IR) index increased (*p* < 0.001) at timepoints 2 and 4 hr and abruptly decreased at timepoint six hr (*p* < 0.01) following LE infusion. In LE group, HOMA-IR values were significantly (*p* < 0.001) higher than those for NS group at timepoints 2 and 4 hr after infusion. Neither treatment nor time influenced serum sodium and potassium concentrations (*p* > 0.05). In conclusion, intravenous infusion of Lipovenoes temporarily influenced some blood constituents. Increased triglyceride concentrations were associated with an increase in HOMA-IR values indicating a state of insulin resistance. No remarkable adverse effect was observed following LE infusion and lipid based emulsions can be safely used in ruminants not suffering from extensive lipid mobilization.

## Introduction

Many critically ill patients suffer from a state of negative energy balance and progressive and/or prolonged forms of this state may result in adverse outcomes and serious complications including increased numbers of infectious problems.^1^ In human patients and animal studies, parenteral nutrition improves wound healing, minimizes muscle protein loss, decreases the weight loss usually seen in catabolic patients, a state of rapid weight loss and loss of fat and skeletal muscle mass, and supports immune function in patients that cannot tolerate oral nutrition.^2^ However, long term use of parenteral nutrition based solely on glucose for supplying the energy in patients with negative energy balance may induce hyperglycemia, essential free fatty acids deficiency, carbon dioxide over-production and liver dysfunction abnormalities.^3^^,^^4^ If prolonged parenteral nutrition is warranted, intravenous lipid supplementation can be used to meet calorie requirements and prevent break-down of protein reserves of body for energy. Lipid is the most calorically dense nutrient, providing 9 kcal g^-1^ of lipid.^2^ Intravenous lipid emulsions (ILEs) are formulated primarily as a source of essential fatty acids for patients requiring parenteral nutrition. These emulsions are composed of neutral, medium to long-chain triglycerides derived from combinations of plant oils (e.g., soybean), egg phosphatides, and glycerin.^5^ In medicine, ILEs are recommended to supply energy and essential fatty acids in patients unable to ingest food orally or when the gastrointestinal tract does not work correctly.^6^

Moreover, currently ILE is recommended as an antidote in cardiovascular and central nervous system sequelae of local anesthetic and non-local anesthetic drug toxicity in medicine.^7^^,^^8^ The ILE has been considered as a novel method for treating local anesthetic systemic toxicity.^9^ ILEs also show promising results as effective antidotes for cardiovascular collapse or cardiac arrest secondary to poisoning or overdose of other highly lipophilic drugs.^9^^,^^10^ In addition, the efficacy of lipid emulsion infusion has been shown for treating overdoses across a wide spectrum of drugs including beta blockers, calcium channel blockers, parasiticides, herbicides and several varieties of psychotropic agents.^9^

In contrast to the available human data, there is very limited information about the application of the ILEs for parenteral nutrition or for the management of toxicities in veterinary medicine. There are some animal (cat, dog, rabbit, and pig) publications in the form of experimental studies.^11^ More recently, ILE therapy has been used in certain poisoning cases in small animal medicine.^5^ However, to the best knowledge of the authors there are no published reports on the use of ILEs, neither as parenteral nutrition nor as an antidote, in ruminant medicine. As a preliminary work, the objective of the present study was to evaluate the effect of intravenous infusion of a soybean based lipid emulsion on some blood constituents in sheep as a model for ruminants.

## Materials and Methods

**Study design. **Four nonpregnant clinically healthy ewes (body weight 45.0 ± 3.0 kg, age 14.0 ± 2.0 month) were used in a two-treatment crossover design to investigate the effects of IV infusion of a soybean based lipid emulsion (Lipovenoes 10% PLR, Fresenius Kabi Deutschland GmbH, Homburg, Germany) on some blood constituents. The individual fatty acid contents of the Lipovenoes are shown in Table 1. Other compositions of this lipid emulsion in one liter volume are glycerin (25 g), phospholipid with 73-80% 3-sn-phosphatidyl choline (6 g), Sodium oleat, Sodium hydroxide and Water.

**Table 1:** Individual fatty acid contents of Lipovenoes ^®^ PLR 10%.

**Fatty Acids**	**% Total FA**
**Palmitic acid** **:** ** C16:0**	10
**Stearic acid** **:** ** C18:0**	4
**Oleic acid** **: ** **C18:1 (n-9)**	24
**Linoleic acid** **: ** **C18:2 (n-6)**	54
**α-Linolenic acid** **: ** **C18:3 (n-3)**	8

The sheep were housed individually in indoor pens and fed adjusted ration to maintain body weight.^12^ The animals had *ad libitum* access to a trace mineral block and water. The overall health of the sheep were monitored before and during the study period. The animals were allowed three weeks to adjust to the diet and handling. The study protocol was approved by the Research Council of Urmia University and the animals were handled and cared for according to the guidelines approved by this Committee. 

The experiment was conducted as a replicated 2 × 2 Latin square design. Treatments consisted of intravenous infusion of lipid emulsion (LE group) or the same volume of normal saline (NS group) as the control, so that each sheep received either NS or the LE in each period with a four week washout interval between periods before switching to the other treatment to avoid carryover effects. 

Two catheters (16-gauge, 3.25-inch) were inserted into the right and left jugular veins of experimental sheep one day before starting the experiment. The left catheter was for infusion of NS or LE and the right one for blood sampling. The catheters were flushed with heparin (10 U of heparin mL^-1^ of saline) at 3 hr intervals. Sheep were fasted overnight before each treatment, but were allowed water *ad libitum.*

The LE or NS were infused at a rate of 0.025 mL kg^-1^ per min for 6 hr using a two-channel infusion pump (Model AP 22; Ascor, Warsaw, Poland). Currently, there was no recommended or published dose for ILE administration in ruminants and we extrapolated the dose from human and small animal data.^5^^,^^13^^-^^16^

**Blood sampling and analytical procedures. **Blood samples were collected via the jugular vein into 4-mL tubes containing potassium oxalate and sodium fluoride for plasma harvesting and plain tubes to remove serum at 0 (baseline), 2, 4, 6, 12 and 24 hr after starting the infusions. Plasma collection was carried out by centrifugation of blood samples at 1600 *g *at 4 ˚C, for 15 min, whereas procedure for serum separation was centrifugation at 2500 *g *at 20 ˚C for 15 min. All plasma and collected serum samples were stored at – 20 ˚C until analysis. Serum insulin was assayed by a commercial ELISA kit (Accubind ELISA Microwells; Monobind Inc. Lake Forest, USA) using an auto-analyzer machine (Biotechnica, Rome, Italy); inter-and intra-assay coefficient of variations (CVs) were 4.3% and 7.2%, respectively. Plasma glucose was measured by enzymatic colorimetric method using glucose oxidase kit (Pars Azmoon Inc., Tehran, Iran); inter-and intra-assay CVs were less than 2.2%. Serum triglyceride, calcium, phosphorous, and magnesium concentrations were measured using commercial kits (Pars Azmoon Inc., Tehran, Iran) according to manufacturer’s instructions. Inter-and intra-assay CVs were 1.6% and 0.6% for triglyceride, 3.0% and 2.4% for calcium, 4.1% and 3.2% for phosphorous, respectively. Serum potassium and sodium concentrations were quantified by Flame photometric method (Model PFP7/C; Jenway, Essex, UK). 

**Estimation the level of insulin resistance. **To predict insulin resistance of the peripheral tissues a surrogate Index, homeostasis model of insulin resistance (HOMA-IR) was calculated according to the following formula:

HOMA-IR=Glucose mmol mL-1×Insulin (μU mL-122.5

The lower the HOMA-IR value, the lower the insulin resistance of an individual.^17^

**Statistical Analyses. **Data were analyzed using SAS (Version 9.3; SAS Institute Inc., Cary, USA). Data were checked graphically to explore for errors and outliers. No outlier was detected. A Repeated Measures ANOVA with PROC MIXED using Kenward-Roger procedure to approximate the denominator degrees of freedom was used for analyses. Model assumptions were assessed visually by examining the distribution of residuals for testing the normality (Q-Q plots) and the homogeneity of variance (using predicted values plotted against residuals to assess). To meet the assumptions, the data for triglyceride and glucose were transformed by applying logarithmic transformation. Treatment, day and their interactions were used as the fixed effects and sheep was considered as random effect. When a non-significant interaction term was detected, the model was re-run with the interaction effect excluded from the model. Different models using the covariance structures including, SP (GAU), SP (POW) and SP (EXP) were compared by the Akaike information criterion (AIC) and the model with minimum AIC was selected. Pair- wise comparisons between least squares means were determined using Bonferroni test. Values are reported as least squares means and SEM. Differences were considered significant when *p* < 0.05.

## Results

**Fig. 1:**
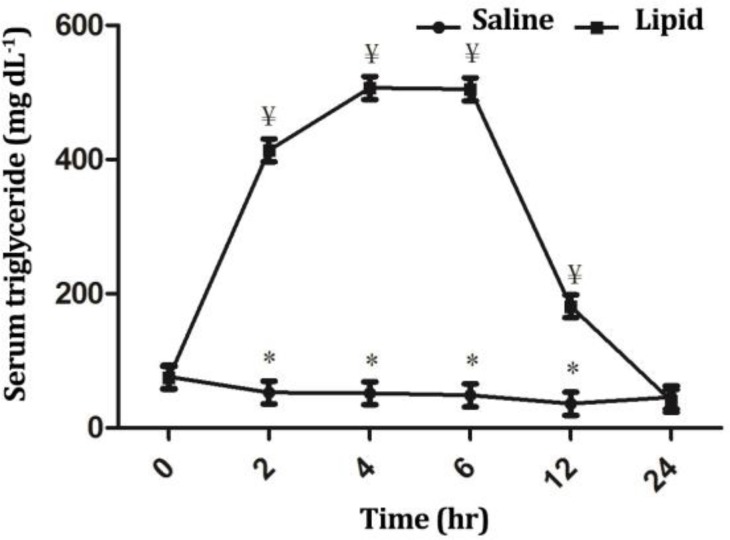
Comparison of the effect of intravenous infusion of a soybean based lipid emulsion and normal saline on serum triglyceride concentrations in sheep. * indicates significant changes between lipid and normal saline infusions at each timepoint, (*p *< 0.05); ¥ indicates significant changes between each time-point compared to baseline value in each group, (*p *< 0.05).

**Fig. 2:**
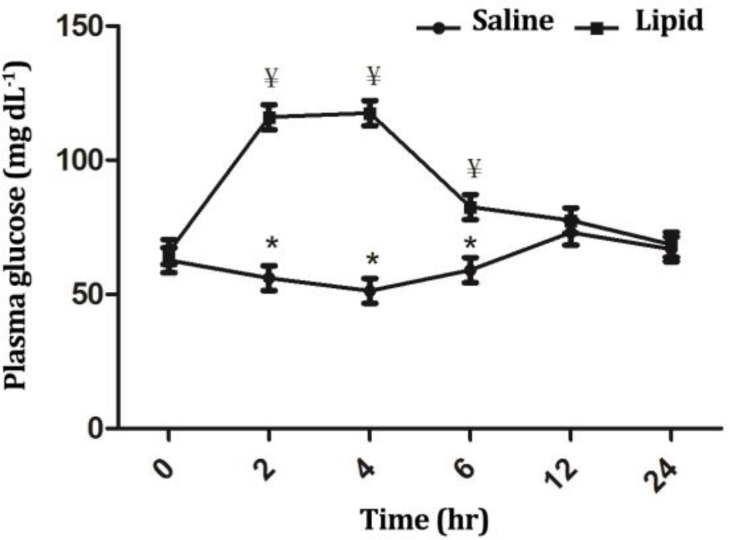
Comparison of the effect of intravenous infusion of a soybean based lipid emulsion and normal saline on plasma glucose concentrations in sheep. * indicates significant changes between lipid and normal saline infusions at each timepoint, (*p* < 0.05); ¥ indicates significant changes between each timepoint compared to baseline value in each group, (*p* < 0.05).

**Fig. 3:**
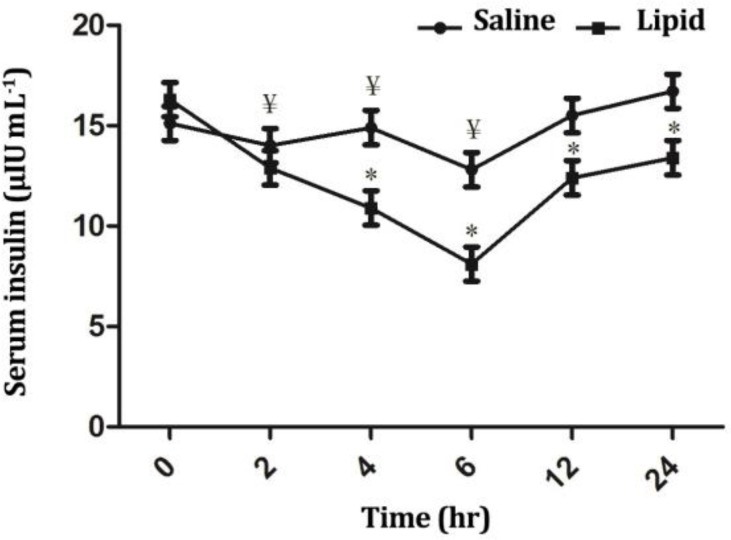
Comparison of the effect of intravenous infusion of a soybean based lipid emulsion and normal saline on serum insulin concentrations in sheep. * indicates significant changes between lipid and normal saline infusions at each timepoint, (*p *< 0.05); ¥ indicates significant changes between each timepoint compared to baseline value in each group, (*p *< 0.05).

Blood triglyceride, glucose and insulin concentrations were influenced significantly (*p* < 0.05) by treatment, time and treatment × time interaction. Infusion of LE elicited a significant increase (*p* < 0.01) in the triglyceride concentration from 2 up to 12 hr after infusion compared to the baseline value (Fig. 1). At these timepoints, triglyceride concentration differed significantly between two groups (*p* < 0.01). Infusion of NS did not significantly influence blood glucose (Fig. 2) and insulin (Fig. 3) concentrations throughout the study; However, LE infusion caused an increase of glucose (*p* < 0.05) and a decrease of insulin levels at timepoints 2 (*p* < 0.05), 4 and 6 hr (*p* < 0.01) compared to the baseline value. Compared to the NS group, in the LE group the concentrations of glucose were increased (*p* < 0.05) from 2 to 6 hr and insulin decreased (*p* < 0.01) from 4 to 24 hr after start of infusion (Figs. 2 and 3).

**Fig. 4:**
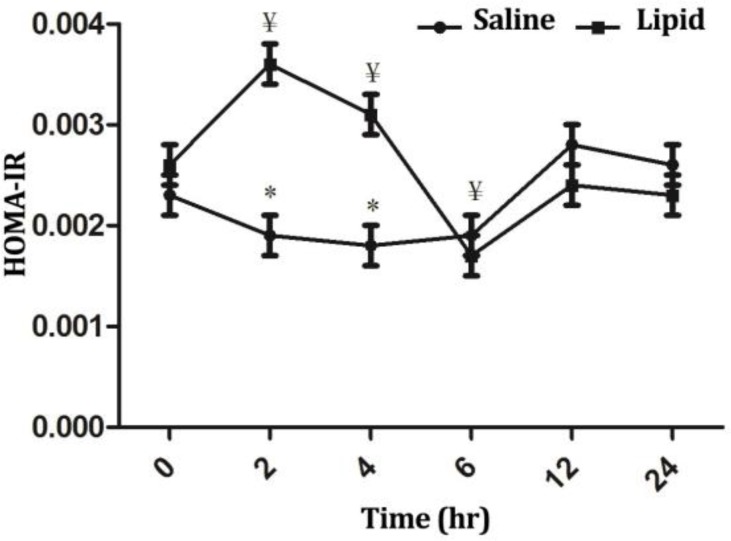
Comparison of the effect of intravenous infusion of a soybean based lipid emulsion and normal saline on HOMA-IR index in sheep. * indicates significant changes between lipid and normal saline infusions at each timepoint, (*p* < 0.05); ¥ indicates significant changes between each timepoint compared to baseline value in each group, (*p* < 0.05).

Values of HOMA-IR were affected significantly by time, treatment and treatment × time interaction. Normal saline infusion was not influenced significantly (*p* > 0.05) in this index throughout the study. However, LE infusion elicited a significant increase (*p* < 0.001) at timepoints 2 and 4 hr and then an abrupt decrease at timepoint 6 hr (*p* < 0.01), afterward the values returned to the baseline value. At timepoints 2 and 4 hr after start of infusion, the HOMA-IR values for LE group were significantly higher than those for NS group (*p* < 0.01), (Fig. 4).

**Fig. 5:**
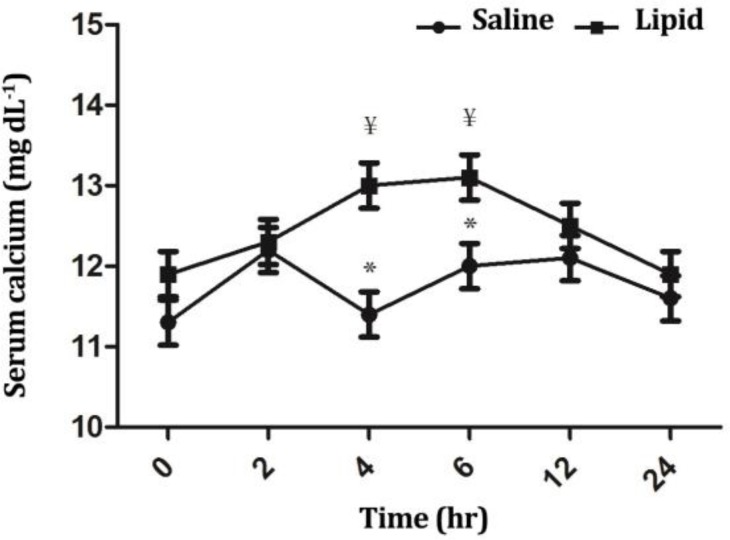
Comparison of the effect of intravenous infusion of a soybean based lipid emulsion and normal saline on serum calcium concentrations in sheep. * indicates significant changes between lipid and normal saline infusions at each timepoint, (*p* < 0.05); ¥ indicates significant changes between each time-point compared to baseline value in each group, (*p* < 0.05).

**Fig. 6:**
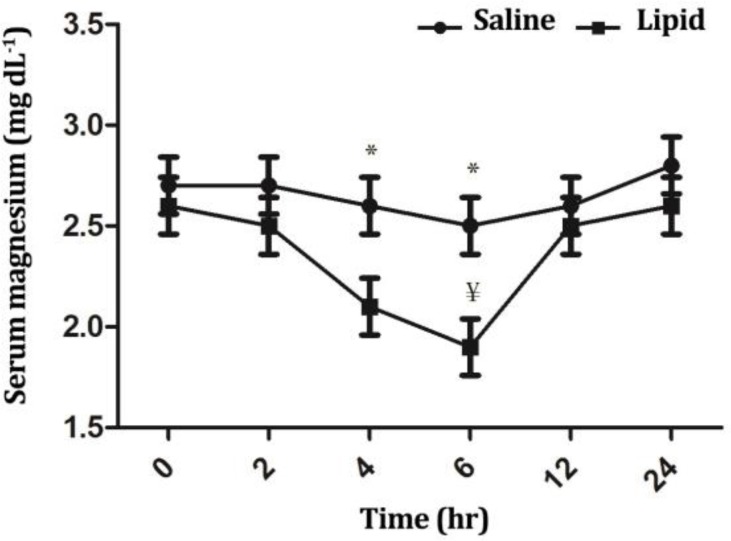
Comparison of the effect of intravenous infusion of a soybean based lipid emulsion and normal saline on serum magnesium concentrations in sheep. * indicates significant changes between lipid and normal saline infusions at each timepoint, (*p* < 0.05); ¥ indicates significant changes between each time-point compared to baseline value in each group (*p* < 0.05).

Serum calcium concentrations were significantly influenced by time (*p* < 0.05), treatment (*p* < 0.0001) and their interaction (*p* < 0.05). In LE group, the levels were significantly higher at timepoints 4 and 6 hr compared to the baseline value (*p* < 0.05). In addition, calcium concentrations were significantly higher for LE group compared to NS group at timepoints 4 and 6 hr (*p* < 0.05), (Fig. 5). The concentrations of serum magnesium were significantly influenced by time (*p* < 0.05) and treatment (*p* < 0.05), but not by their interaction (*p* > 0.05). In LE group, the magnesium levels were decreased (*p* < 0.05) at timepoint 6 hr compared to NS group. Infusion of LE decreased (*p* < 0.05) magnesium concentrations at time-points 4 and 6 hr compared to NS infusion (Fig. 6). There were significant differences in the serum phosphorous concentration over the time of infusion between the two groups (*p* < 0.01). 

**Fig. 7:**
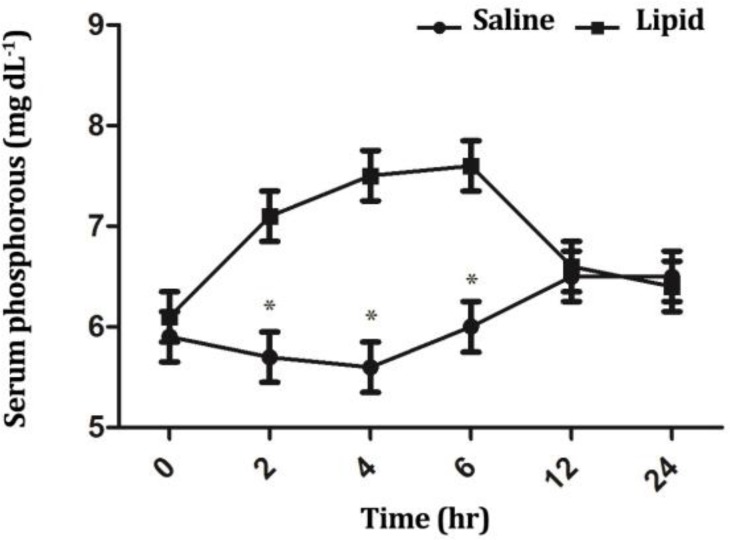
Comparison of the effect of intravenous infusion of a soybean based lipid emulsion and normal saline on serum phosphorous concentrations in sheep. * indicates significant changes between lipid and normal saline infusions at each time-point, (*p *< 0.05).

The LE group had significantly higher mean phosphorous concentrations compared to NS group at timepoints 2, 4 and 6 hr (*p* < 0.05), (Fig. 7). Neither treatment nor time had a significant influence on serum sodium and potassium concentrations (*p* > 0.05).

## Discussion

The effects of intravenous infusion of LE on blood metabolites and minerals have not been previously reported, neither in healthy nor in diseased ruminants. The results of this study indicated that in healthy sheep some temporary changes can be induced by intravenous infusion of LE in some blood constituents levels, however, the values returned to their baseline values up to 24 hr after infusion. 

There is no established dose for ILE administration in ruminants. The general recommended dose for parenteral nutritional therapy in medicine for ILE administration ranges from 0.4 to 4.0 g kg^-1^ per day in children (0.083-0.83 mL kg^-1 ^per hr) to 2 g kg^-1^ per day in adults (0.42 mL kg^-1 ^per hr).^11^ When using a 10% ILE, these dosages represent a volume of 0.17-1.70 mL kg^-1 ^per hr or 0.003 - 0.03 mL kg^-1 ^per min. Such dosages were also recommended in veterinary medicine mainly for small animals, i.e., dogs and cats.^5^^,^^13^^-^^16^ Based on these data we used 0.025 mL kg^-1 ^per min in our experiment.

Intravenous infusion of LE caused an approximately seven fold increase in blood triglyceride levels after infusion and the significant increased levels lasted up to six hr after termination of infusion (12 hr after start of infusion). The observed increased level of serum tri-glyceride in sheep in this study was similar to that showed by Jufri *et al*. in rabbit.^18^ They found that the maximum triglyceride level was achieved four hr after infusion of palm-oil based lipid emulsion and it was shown that the triglyceride concentration returned to baseline level after 6 hr of infusion. However, in our study serum triglyceride concentration remained in high levels for a longer time. This discrepancy may be attributed to the more rapid clearance rate of palm-oil based lipid emulsions compared to soybean based lipid emulsions.^19^

Plasma glucose concentrations increased significantly 2 to 6 hr after infusion of LE. This observation was in agreement with the study of Chikenji *et al*. who found increased serum glucose concentration during infusion of 10% soybean emulsion in normal humans.^20^ This rise in plasma glucose concentration during lipid infusion can be attributed to decreased utilization of glucose by peripheral tissues.^21^ Thiébaud *et al*. indicated that the inhibitory effect of free fatty acids (FFAs) on glucose utilization involves the biochemical pathways regulating both glucose oxidation and glycogen synthesis.^22^ Furthermore, FFAs can increase plasma glucose concentration through the stimulation of gluconeogenesis by several mechanisms including enhancing gene expression of the gluconeogenic enzymes, promoting the hepatic FFAs oxidation leading to increased production of NADH, acetyl-CoA and ATP and inducing a state of insulin resistance.^23^

In LE group, a decrease in insulin concentration was observed and insulin levels remained lower than baseline value for six hr and lower than NS group for 24 hr. In a study in periparturient dairy cows, Kerestes *et al*. demonstrated that a higher concentration of non-esterified fatty acids (NEFAs) was significantly correlated with a lower insulin secretion.^24^ Other studies also demonstrated a decreased insulin-secretory capacity of the pancreas by increased levels of NEFAs in dairy cows. Bossaert *et al*. also reported a negative impact of elevated NEFA levels on the insulin-secretory capacity of the pancreas in dairy cows.^25^ In addition, in ketonemic and starved cows administration of an intravenous bolus of glucose has been associated with lower insulin secretion.^24^^,^^26^ Therefore, it is probable that, in our study increased concentrations of FFAs following infusion of LE resulted in a decreased secretion of insulin from pancreas. However, a state of insulin resistance was also probable in LE group. Insulin resistance could be attributed to a decrease in insulin responsiveness, a decrease in insulin sensitivity or both.^17^ Studies in nonpregnant and nonlactating dairy cow have demonstrated that the elevation of circulating NEFAs following a fasting period^27^^,^^28^ or by intravenous administration of a tallow infusion ^29^ resulted in insulin resistance and an impaired insulin-stimulated glucose uptake by insulin-sensitive tissues. Analysis of the index of insulin resistance indicated that the HOMA-IR values for LE group were significantly higher than those for baseline and the NS group values. This finding would suggest a state of insulin resistance and decreased glucose clearance by tissues. Thus, it seems that increased concentration of FFAs following infusion of LE had a dual influence on blood glucose concentrations, first by decreasing insulin secretion from pancreas and second by inducing a state of insulin resistance. The cause of abrupt decline in HOMA-IR value at timepoint 6 following LE infusion is unclear. Further, detailed studies using sophisticated tests, e.g., hyper- insulinemic euglycemic clamp test or intravenous glucose tolerance test are needed to measure insulin sensitivity more appropriately in ruminants. This is the focus of ongoing study at Urmia University.

Intravenous infusion of LE resulted in a significant increase in serum calcium concentration within the last two hr of infusion compared to baseline value and NS group. Due to presence of a complex, efficient and precise homeostatic mechanism for regulating blood calcium concentration it is difficult to explain this finding in the present study. Increased levels of essential free fatty acids including, Linoleic acid (LA) and α-linolenic acid (ALA) can enhance both calcium absorption from the gut and reduce calcium excretion via the urine.^30^

Since 64.0% of total FFA from LE was LA and ALA, the temporary rise in serum calcium concentration may be associated with increased EFAs and their actions on calcium metabolism. In addition, a significant positive correlation between blood calcium and glucose both before and after calving has been observed in dairy cows.^31^ As explained earlier, in LE group blood glucose concentrations were significantly higher than those of NS group. Therefore, it seems that there is a link between high glucose and calcium levels in LE group in the present study. 

As indicated previously, elevation of plasma FFAs can produce insulin resistance in many tissues. In patients with type II diabetes mellitus, an inverse association exists between the plasma magnesium and insulin resistance due to intracellular shift of magnesium.^32^ In the present study, serum magnesium concentration in LE group at time point six hr decreased to 1.9 mg dL^-1^, which was lower than minimum reference range of magnesium for sheep (2.2 mg dL^-1^).^33^ Transitory insulin resistance due to increased levels of FFAs may be the cause of this temporary hypomagnesaemia in this study.

We observed higher levels of serum inorganic phosphorous following intravenous infusion of LE compared to NS infusion. Our finding was in agreement with Vernon *et al*. who demonstrated an increased concentration of blood phosphorus following LE infusion.^34^ They concluded that phospholipid content of LE (approximately 1.2% of all commercially available lipid emulsions is egg phosphatide, primarily phosphatidyl choline) was the cause of increased inorganic phosphate pool following LE infusion. In another study, the effects of ILE on the serum phosphorus concentration in the 16 ICU postoperative patients were investigated.^35^ Results of that study showed that serum phosphorus concentration was significantly increased in 11 ICU patients after infusion. 

There was no effect of NS or LE infusions on serum sodium and potassium concentrations. In agreement with our results, Pelikánová *et al*. demonstrated that acutely induced hypertriglyceridemia did not alter renal hemodynamics or renal sodium handling.^36^

Some reports highlighted the side effects of ILE administering in large doses. Increased risk of complications including, pulmonary or neurological complications,^9^ oxidative stress, alterations in cell-mediated immunity, inflammation and thrombosis ^1^ have been attributed to ILE administration. The sheep used in this experiment were monitored periodically up to six months after intervention and no clinical complications have been observed. However, some periparturient diseases in ruminants, like hepatic lipidosis in cattle and sheep, are associated with extensive lipolysis and lipid mobilization. In these conditions, administration of ILEs may exacerbate the problem and lipid based products should not be used in such cases. However, it seems that, short-term administration of these products can be safely used in ruminants not suffering from extensive lipid mobilization.

The small sample size and using only a single dose administered for six hr, were the major shortcomings of the present study. More detailed animal studies using randomized controlled trials with sufficient sample sizes in animals with different clinical conditions and with various dosage regimens are needed for evaluating the usefulness and potential side effects of ILEs in ruminants.

In conclusion, short-term intravenous infusion of soybean based lipid emulsions influenced blood triglyceride, insulin, glucose, calcium, magnesium and phosphorous concentrations. The changes in the levels of these variables were transitory and were mainly within their physiologic limits. It seems that induction of a state of insulin resistance is the mainstay of these changes. 
